# TNF-α and IFN-γ gene variation and genetic susceptibility to type 1 diabetes and its microangiopathic complications

**DOI:** 10.1186/2251-6581-13-46

**Published:** 2014-04-01

**Authors:** Javad Tavakkoly Bazzaz, Mahsa M Amoli, Zahra Taheri, Bagher Larijani, Vera Pravica, Ian V Hutchinson

**Affiliations:** 1 Department of Medical Genetics, School of Medicine, Tehran University of Medical Sciences, Tehran, Iran; 2 Endocrinology and Metabolism Research Centre, Tehran University of Medical Sciences, Tehran, Iran; 3 School of Pharmacy, University of Southern California (USC), Los Angeles, USA

## Abstract

**Background:**

TNF-α has accelerating role in development of type 1 diabetes. Although an immunosupressor function and leading protecting role in T1DM also has been claimed for this pro-inflammatory cytokine. Over-expression of pro-inflammatory and type 1 cytokines (Th1, like IFN-γ) drive insulitis toward the destructive form that leads to type 1 diabetes (T1DM). Among type 1 cytokines only IFN-γ has been detectable in the islet β cells. In deletion studies IFN-γ was also the only Th1 cytokine for which its ablation or blockade caused delayed or decreased incidence of T1DM.

**Methods:**

Functional polymorphisms of TNF-α at position -308*G/A and at position +874*T/A of IFN-γ gene were employed as markers and the comparative distribution of derived genotypes/alleles were assessed in 248 British Caucasian T1DM patients and 118 healthy controls.

**Results:**

There was no significant association between IFN-γ gene polymorphism and T1DM or the diabetic complication triad. There was a marginal association between TNF-α –308*G/A polymorphism in nephropaths (vs healthy controls) (p = 0.06), which its insignificancy may be due to survivor factor. No significant association was evident between the genotype/allele of the applied marker and T1DM or diabetic complication triad.

**Conclusion:**

Our results are in contrast with previous reports suggesting that these polymorphisms are not related to T1DM. This study also underlines the importance of replication of association studies to confirm the previous interpretation.

## Introduction

TNF-α gene in human is a single copy gene and is located on the short arm of chromosome 6 in close linkage with MHC genes [1].

TNF-α is a potent inflammatory mediator, which is produced by monocytes, macrophages, CD4+ and CD8+ T cells, B cells, lymphokine-activated killer (LAK) cells, NK cells, endothelial cells, a number of non-haematopoietic tumour cell lines and also other sources such as mast cells and neutrophils upon stimulation [2,3]. General expression of TNF-α receptors by a wide variety of cells and tissues suggests that TNF-α is involved in a number of biological activities [4]. Besides its pro-inflammatory role, TNF-α has other functions such as promotion of T cell proliferation in vitro [5,6], prevention of T cell deletion induced by superantigens [7], and it critically influences germinal centre formation following immunization [8]. In pathological circumstances, all of these properties, which contribute to the establishment, maintenance, or accentuation of specific immune responses, could aberrantly end in tissue injury [4].

Interferon-gamma (IFN-γ), also known as type II interferon or macrophage-activating factor (MAF), was originally identified due to its antiviral activity. The mature IFN-γ protein has a molecular weight about 17 kDa [9].

Both genetic and environmental factors contribute to the onset of T1DM [10] and its chronic complications. Genetic susceptibility to T1DM probably includes an inherited defect in the establishment of peripheral tolerance to β cell autoantigens. One of the most important genetic risk factors is inheritance of particular NOD MHC class II alleles [11,12].

The critical role of TNF-α in development of many inflammatory and autoimmune diseases has encouraged researchers to pursue the influence of different TNF-α gene polymorphisms on development of some diseases including T1DM by a series of association studies. While TNF-α has also metabolic potentials, the impact of its polymorphisms on glucose dysmetabolism and related consequences has not been studied yet.

TNF-α gene polymorphisms and their impacts on the level of TNF-α production and diseases have been reviewed [1].

It is well documented that the destructive form of insulitis is associated with over-expression of pro-inflammatory (IL-1, TNF-α, and IFN-α) and type 1 cytokines (IFN-γ, TNF-β, IL-2 and IL-12), whereas the up-regulation of type 2 (IL-4 and IL-10) and type 3 cytokines (TGF-β1) are reported in benign insulitis, reflecting the role for cytokines as regulators as well as mediators of immune responses. Also, in deletion studies of type 1 cytokines, the protecting effect of cytokine ablation was exclusive for IFN-γ. It has been observed that the knocking out of the IFN-γ gene, IFN-γ neutralisation, IFN-γ blockade, or deletion of IFN-γ-R positive cells in NOD mice and BB rats all led to delayed or decreased incidence of T1DM [13].

The human IFN-γ gene is located on chromosome 12, spaning 6 kb in length and contains four exons and intermediate introns [14].

The IFN-γ polymorphism at position +874*T/A in the first intron is correlated with the level of the IFN-γ production, where allele T is the high producer. This polymorphism coincides with a putative NF-κB binding site that may mediate high production of IFN-γ [15]. The previously reported CA repeat polymorphism in the first intron [16] of which allele 2 (12 CA repeats) was the producer of higher level, is immediately adjacent and absolutely correlated with allele T of the polymorphism at position +874 [17].

In present study genetic susceptibility to T1DM and its chronic complications have been monitored through a potentially shared contributor, TNF-α and IFN-γ. The frequency of the TNF-α gene polymorphism at position -308*G/A, which influences the transcriptional activation [18] and the level of its expression [19-21] besides IFN-γ polymorphism at position +874*T/A was examined in 248 Caucasian T1DM patients and 119 healthy controls.

### Patients and controls

In this cross-sectional study, in total 248 unrelated British Caucasian with T1DM were randomly selected among patients attending Manchester Diabetes Centre during 1999–2002. The ethical approval was obtained from the Manchester Royal Infirmary.

All patients fulfilled the relevant criteria for related diagnosis as are detailed below. To be on the safe side, patients who had diabetes less than 3 years were excluded from analysis.

### Type 1 diabetes

Diabetes was diagnosed according to the criteria, which was suggested by an expert committee in 1997 (report of the expert committee on the diagnosis and classification of diabetes Mellitus, 1997). The diabetic patients included in the present study fulfilled at least one of the triple criteria recommended by the expert committee, detailed as follows:

a) Symptoms of hyperglycaemia (polyuria, polydipisia, unexplained weight loss) plus random plasma glucose > 200 mg/dL (11.1 mmol/L). Random is defined as any time of day without regard to time since last meal.

b) Fasting plasma glucose (FPG) > 126 mg/dl (7.0 mmol/L). Fasting is defined as no caloric intake for at least 8 hours.

c) 2-hour plasma glucose (PG) > 200 mg/dl (11.1 mmol/L) during an oral glucose tolerance test (OGTT). The test should be performed as described by the World Health Organization (WHO), using a glucose load containing the equivalent of 75-g anhydrous glucose dissolved in water.

Diabetes was regarded as T1DM if it was diagnosed before age of 30 years and accompanied with acute onset and treatment with insulin began within the first year of diagnosis and continued thereafter.

### Diabetic retinopathy (DR)

The back of the eye was examined by fundoscopy (after pupillary dilatation) and when more than five dots or blots per eye, hard or soft exudates or new vessels were evident the diagnosis of retinopathy was applied. Patients who had a history of laser treatment were also diagnosed as retinopathy.

### Diabetic nephropathy (DN)

The elevation of AER (> 300 mg-2 g/day) at least on two of three occasions and/or 3 positive Albustix over the past 12 months were evident to mark patients as nephropath, while a urinary tract infection (UTI) was ruled out already.

### Diabetic neuropathy (DNU)

Neurothesiometer -a clinical electromagnetic vibrating device- was applied for screening of peripheral diabetic neuropathy, which quantifies the vibration sensitivity through the measurment of vibratory perception threshold (VPT), while the patient’s eye is closed and the probe of neurothesiometer is placed on the hallux of the toe. An average of 3 readings were taken. DNU was diagnosed when VPT was more than 25 volts (vibration threshold above 25 volts indicates a high risk of ulceration). The symptoms of sensory and/or motor neuropathy were looked for, like parasthesia, numbness, tingling, nocturnal rest ache, all in the absence of peripheral vascular disease as non-specific (non-diabetic) underlying cause. The excluding of peripheral vascular disease was approved by palpable pulses and measuring of ankle brachial pressure index.

## Materials and methods

The ARMS-PCR method was applied for genotyping of TNF-α -308*G/A polymorphism and IFN-γ +874*T/A polymorphism. The sequences of designed primers were illustrated in Table 1.

**Table 1:** Primer sequences for TNF-α and IFN-γ gene polymorphisms genotyping

**Internal control primers (Human growth hormone)**		
HGH1 5′- GCCTTCCCAACCATTCCCTTA-3′		PCR product size 425 bp
HGH2 5′- CAAGGATTTCTGTTGTGTTTC-3′		
**Gene specific primers**		
TNF-α (-308*A/G)	5′-TCTCGGTTTCTTCTCCATCG-3′	PCR product size 184 bp
	5′-ATAGGTTTTGAGGGGCATGG-3′	
	5′-AATAGGTTTTGAGGGGCATGA-3′	
IFN-γ (+874*T/A)	5′-TCAACAAAGCTGATACTCCA-3′	PCR product size 262 bp
	5′-TTCTTACAACACAAAATCAAATCT-3′	
	5′-TTCTTACAACACAAAATCAAATCA-3′	

A master mix solution was used for DNA amplification. This master mix includes 22% of Ready Load Reaction Buffer (AB Technology, UK), 22% of 200 μM dNTPs (AB Technology, UK), 13% of 1.5 mM magnesium chloride (AB Technology, UK), 31% of 60% (W/V) sucrose, 11% internal control primer (1 μM each), and 1.1% of Thermoprime DNA Polymerase (AB Technology, UK). For each sample, 1.5 μl of DNA was added to 15 μl of the master mix solution and then 5 μl of specific primer mix was aliquoted to 5 μl of master mix solution, which already contains DNA as well. After that, this 10 μl reaction was amplified on a PTC-100 PCR thermal cycler (MJ Research, Inc), where the cycles was programmed as follows: 1 minute at 96°C followed by 10 cycles of 15 seconds at 95°C, 50 seconds at 65°C for TNF-α and 62°C for IFN-γ 40 seconds at 72°C followed by 20 cycles of 20 seconds at 95°C, 50 seconds at 59°C and 50 seconds at 72°C.

In gel electrophoresis, according to the presence or absence of amplified targeted sequence, the type of alleles (genotype) were identified. The amplified products were visualized on a 2% agarose gel against 200 bp ladders and stained with 5 μl (0.5 mg/ml) of ethidium bromide.

## Results

**Figure 1:**
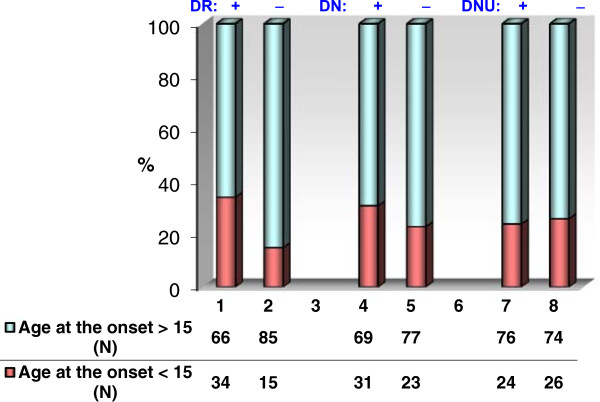
Microvascular diabetic complications according to the age at the onset of diabetes (under or over 15 years).

None of the diabetic late complications were significantly correlated with the gender (p > 0.05). Among triad of diabetic complications only in DR group the impact of age at the onset > 15 years was statistically determinant (p = 0.0008) for the development DR. The impact of the age at the onset was insignificant for the development of both DN (p = 0.2) and DNU (p = 0.7) (Figure 1). Please insert Figure 1 (as follow).

No significant differences were evident in the distribution of IFN-γ +874*T/A and TNF-α -308*G/A polymorphism among cases, controls and different subgroups. Tables 2 and 3.

**Table 2:** Distribution of genotype and allele frequencies of the IFN-γ +874*T/A polymorphism in healthy controls (C), type 1 diabetic subjects (P), diabetic retinopaths (DR), nephropaths (DN), neuropaths (DNU) and complication free (CF) group*

IFN-γ +874*T/A	**C n (%)**	**P n (%)**	**DR n (%)**	**DN n (%)**	**DNU n (%)**	**CF n (%)**
**Genotype**						
**TT**	26 (22.0)	54 (22.0)	27 (20.0)	18 (21.0)	17 (21.0)	23 (23.5)
**TA**	62 (52.0)	128 (51.0)	70 (52.0)	45 (52.0)	43 (52.0)	49 (50.0)
**AA**	31 (26.0)	66 (27.0)	38 (28.0)	23 (27.0)	22 (27.0)	26 (26.5)
**Allele**						
**T**	114 (48.0)	236 (47.6)	124 (46.0)	81 (47.0)	77 (47.0)	95 (48.5)
**A**	124 (52.0)	260 (52.4)	146 (54.0)	91 (53.0)	87 (53.0)	101 (51.5)

*There were no significant differences between controls and diabetic patients or between patients with or without diabetic complications.

**Table 3:** Distribution of genotype and allele frequencies of the TNF-α at -308*G/A polymorphism in healthy controls (C), T1DM subjects (P), diabetic retinopaths (DR), nephropaths (DN), neuropaths (DNU) and complication free (CF) group*

**TNF-α** -308*G/A	**C n (%)**	**P n (%)**	**DR n (%)**	**DN n (%)**	**DNU n (%)**	**CF n (%)**
**Genotype**						
GG	73 (62.0)	136 (54.8)	75 (55.5)	44 (51.2)	46 (56.1)	54 (55.1)
GA	39 (33.0)	91 (36.7)	49 (36.3)	33 (38.4)	29 (35.4)	36 (36.7)
AA	6 (5.0)	21 (8.5)	11 (8.2)	9 (10.5)	7 (8.5)	8 (8.2)
**Allele**						
G	185 (78.4)	363 (73.2)	199 (73.7)	121 (70.4)	121 (73.8)	144 (73.5)
A	51 (21.6)	133 (26.8)	71 (26.3)	51 (29.6)	43 (26.2)	52 (26.5)

*No significance difference was evident between any groups/subgroups.

## Discussion

TNF-α as a pro-inflammatory cytokine is crucial contributor in development of T1DM, as its functional disruption by specific lack of TNF-α-R1 leads to profound protection from autoimmune diabetes [22]. However, during T cell mediated autoimmunity TNF-α as a Th1 cytokine exacerbates the evolvement of autoimmune diseases like T1DM [23]. In contrast it can act as an immunosuppressor as well. The time and site of exposure to TNF-α determine the outcome [24]. When it is injected in NOD mice before 4 weeks of age [25] or expressed transgenically at β islets in neonatal NOD mice [26] it promotes the course of autoimmune reactions and predisposes the animal to T1DM. On the contrary, its late, transgenic expression leads to dampening of auto-reactive T cell repertoire and protection from diabetes [27].

The diabetogenic impact of TNF-α in transgenic NOD mice is ascribed to the early recruitment and activation of Dendritic cells and macrophages and, then by reinforcement of self-antigen uptake and presentation and not via the direct effect on β cells [26]. While immuno-suppressory role of TNF-α may be attributable to its promoting effect on T cell apoptosis in turning off unnecessary immune responses [28] or attenuating its impact on TCR signal transduction pathways due to chronic stimulation with TNF-α [29].

Due to possessing of another range of functions (other than pro-inflammatory) mainly suppression of insulin signalling, TNF-α can be regarded yet again as a candidate in aethiopathogenesis of diabetic late complications. However, this dual functionality of TNF-α in backward-looking analysis (such as the present cross sectional study) cannot be distinguished from each other as any association between TNF-α genotype and late complications may be secondary to a primary association between TNF-α and T1DM itself. But the diabetic subjects who are free from particular complications could be regarded as intermediary controls to compare with each complication that could minimize the bias tic impact of previous association.

The polymorphism of TNF-α gene at position -308*G/A is reportedly correlated with transcriptional activation [18] and its functionality has been described. While some reports claimed allele A is the higher producer variant [18,20], in contrary allele G also was found as the high producer [19,21] as well.

Since our data did not demonstrate any significant association between TNF-α–308*G/A polymorphic genotypes/alleles with T1DM or its complications, however there was a consistent increase in frequency of allele A among patients at each category relative to healthy controls. In respect to controversial findings mentioned above such increased frequency of allele A in type 1 diabetics as a whole can be interpreted in two ways. Either the allele A is the high producer or the low producer variant, and because TNF-α can play a dual role, both promoter and dampener in development of T1DM. But with reference to the steady increased frequency of allele A throughout other categories (different complications) where the elevated level of TNF-α might be favored in their development, in overall these may confer that allele A is possibly the high producer variant (at least in the case of diabetic context).

The greatest difference in distribution of polymorphic alleles was evident in the diabetic nephropath subgroup, where the significance was marginal (p = 0.06). The distinctiveness of the nephropath subgroup can be explained by a stronger role of TNF-α in development of DN relative to two other complications. It may also be due to more influential capacity of genetic issues in development of DN rather than DR and DNU.

Furthermore, since DN is the only complication among the three complications which is followed by mortal outcome, the previous removal of a fraction of nephropaths by death, who should had carried the risky genotypes more frequently than the survivor nephropaths may have biased the present results. If all patients with nephropathy were taken into account, i.e. by prospective (follow-up) study, the marginal difference possibly will reach the significant level. All these considerations urge the present study should be repeated in independent group of patients reminding that the appropriateness of our interpretation in case–control studies basically depends on reproducibility of the results in different group of patients.

The distribution of the polymorphic alleles also was compared between diabetic nephropaths and diabetic subjects without DN and the difference was not significant. Likewise there was no significant difference in frequency of those alleles neither between diabetic subjects with and without DR and those with and without DNU.

The frequency of allele A in diabetics who were free from the triad of late complications was similar to other subgroups (except DN) and to diabetics as a whole. Such sameness in allele frequency may firstly criticize the soundness of patients’ stratification in complication free group, as they were placed in a group by the “lack” of too broad manifestations (different complications). Otherwise, it may imply that in terms of diabetic complications this polymorphism is not reliable/capable to partition the diabetic subjects, while it may do so in combination with other polymorphic markers (in the same or nearby genes) and by adding the number and power of detecting mechanism.

While the non-significant role of TNF-α gene polymorphism in development of T1DM in the present study is consistent with the result of another study, conducted in US Caucasian and Chinese diabetics living in Taiwan [30], following points can be raised as concluding remarks.

Although TNF-α may have opposing accelerating/protecting effects in development of T1DM; we anticipated a linkage between TNF-α and T1DM and with its late complications. However, association studies can give false results. Small numbers of cases/controls, lack of homogeneity in cases/controls population, uncertainty in clinical definitions and inexactness in patients’ selection, selection of the wrong SNP (other SNP rather than -308) and other inconsistencies may contribute to false negative results. For instance while the present study has 90% power to detect an association with an odds ratio (OR) of 3 or more, the sample size is clearly underpowered to detect an association with an OR less than 2, which may be the anticipated level of association in multigenic complex traits. On the other hand, a multivariate trait like diabetic complications can be produced or be influenced by different proportional contribution of genetic vs environmental factors. For example, in order to reduce the number of variables we can categorize the patients’ group according to the duration of diabetes at the onset of each complication that eliminates the impact of such a major environmental factor and places patients in phenotypically more homogeneous sub-groups, which in turn requires a higher number of patients to reach statistical power.

IFN-γ seems to be extremely important in susceptibility and progression of diabetes. In this study there was also no significant association between IFN-γ gene polymorphism at position +874*T/A and diabetes or its chronic microvascular complications The lack of association between the +874*T allele and diabetes may suggest that this polymorphism is not functional in the context of diabetes. Other polymorphisms may be of importance in β cell destruction.

The main intent to select IFN-γ as a candidate was to explore its effect in the development of diabetes rather than its complications. Although the selected polymorphism had advantageous criteria (functionality, high frequency of both alleles), the number of patients was reasonably high and well-defined diagnostic criteria were employed to characterize patients, it seems that the likelihood to interpret our findings as a false negative result due to a methodological problem is low. However, in two independent studies associations of IFN-γ polymorphisms with T1DM have been reported. One polymorphism was the CA repeat polymorphism located within the first intron [31] and the other in the promoter region at position -590*C/T [32]. The high producer allele of CA repeat polymorphism is the same as the +874*T allele genotyped in this study. The inconsistency of association may be due either to differences in the location of the examined polymorphism (-590 compared with +874), or in the case of the same polymorphism, may be due to irregularity (presence/absence) of linkage disequilibrium between the examined allele and the causative allele in close proximity among different subjects (population admixture). It is obvious that if the examined polymorphism was the very allele linked to diabetes, it should demonstrate constant association in all instances, even in stratified populations.

Similar to other cytokines, IFN-γ may retard or accelerate the course of T1DM development, according to the dose, frequency, site and timing of its administration, and also the species of diabetes-prone animal model (NOD mouse or BB rat). For example, while the local expression of IFN-γ has a tolerance breaking and diabetogenic effect in Balb/C mice, in NOD mice this expression has a protective effect. IFN-γ, in addition to selective inhibitory effects on Th2 cells proliferation, has an intricate role in antiviral host defense mechanisms and enhancement of inflammatory reactions.

The pro-inflammatory role of IFN-γ in exacerbation of autoimmune responses can be considered at different levels. One of the main mechanisms is up-regulation of MHC expression on both professional APCs [33] and the cells under attack [34]. IFN-γ can also influence the processing and presentation of self-antigens in target organs by macrophages and DCs [35].

The exact mechanisms for an anti-inflammatory role of IFN-γ are not clearly pointed out. IFN-γ can either directly [36] or indirectly (via triggering of Bcl2-mediated apoptosis) [37] suppress autoreactive T cells. It also has been reported that the cell death of activated autoreactive T cell is induced by anti-TCR mAbs (as mimic ligands) in the absence of co-stimulatory cells which has been proposed as the third mechanism of tolerance besides intra-thymic deletion of developing autoreactive T cells and peripheral inactivation of mature, naïve T cells [38].

The lack of association is not surprising for diabetic complications, where IFN-γ cannot be considered as a pivotal mediator. But with regard to the crucial role of IFN-γ in the development of diabetes these findings may highlight the bi-directional (pro/anti) impact of IFN-γ in the development of T1DM. While the dynamic actions of IFN-γ (namely in time and site-dependent manner) in terms of T1DM anticipates different outcomes, it seems such complexity (relativity) may not be deciphered by the association test which examines the absolute role of a given polymorphism in a very large phenotypic entity (disease).

Repeating this study collecting higher number of samples and polymorphisms covering entire length of the gene the power of the study will be increased. Dividing patients into groups according to their clinical data, namely the duration of diabetes at the time of complication onset, and the severity of each complication, makes them more homogeneous and reduces the number of confounding factors bringing the light into the role of genetic factors in disease modification.

## Conclusion

The irreproducibility of results in association studies in a disease may reflect its stochastic nature. It worth drawing attention to the fact that just 6 of 166 positive associations that have been reassessed three or more times have been able to reproduce same results. It is concerning also that among over 600 reported positive associations, over 400 of those have not yet been replicated more than twice and still are on the waiting list for further evaluation [39].

However, despite the acknowledged limitations of association studies, they have been particularly recommended in genetic dissection of complex traits [40], regardless of the fact that they can only give suggestive clues rather than absolute proof.

## Competing interests

The authors declare that they have no competing interests.

## Authors’ contributions

JTB conceived the study, collected the samples, carried the molecular genetic study and drafted the manuscript, MMA drafted the manuscript, ZT drafted the manuscript, BL gave valuable advise which helped in drafting the manuscript, VP conceived the study, IV was principle investigator and conceived the study. All authors read and approved the final manuscript.
